# Origin of Nanobubbles Electrochemically Formed in a Magnetic Field: Ionic Vacancy Production in Electrode Reaction

**DOI:** 10.1038/srep28927

**Published:** 2016-07-05

**Authors:** Ryoichi Aogaki, Atsushi Sugiyama, Makoto Miura, Yoshinobu Oshikiri, Miki Miura, Ryoichi Morimoto, Satoshi Takagi, Iwao Mogi, Yusuke Yamauchi

**Affiliations:** 1 National Institute for Materials Science, Tsukuba, Ibaraki 305-0044, Japan; 2 Polytechnic University, Sumida-ku, Tokyo 130-0026, Japan; 3 Research Organization for Nano and Life Innovation, Waseda University, Shinjuku-ku, Tokyo 162-0041, Japan; 4 Hokkaido Polytechnic College, Otaru, Hokkaido 047-0292, Japan; 5 Yamagata College of Industry and Technology, Matsuei, Yamagata 990-2473, Japan; 6 Yokohama Harbor Polytechnic College, Naka-ku, Yokohama 231-0811, Japan; 7 Saitama Prefectural Showa Water Filtration Plant, Kasukabe, Saitama 344-0113, Japan; 8 Koriyama Technical Academy, Koriyama, Fukushima 963-8816, Japan; 9 Institute for Materials Research, Tohoku University, Sendai, Miyagi 980-8577, Japan

## Abstract

As a process complementing conventional electrode reactions, ionic vacancy production in electrode reaction was theoretically examined; whether reaction is anodic or cathodic, based on the momentum conservation by Newton’s second law of motion, electron transfer necessarily leads to the emission of original embryo vacancies, and dielectric polarization endows to them the same electric charge as trans- ferred in the reaction. Then, the emitted embryo vacancies immediately receive the thermal relaxation of solution particles to develop steady-state vacancies. After the vacancy production, nanobubbles are created by the collision of the vacancies in a vertical magnetic field.

In electrolysis in aqueous electrolyte solution, gas evolutions of hydrogen and oxygen often occur^1^,^2^. Whenever electrode potential is set more cathodic or anodic beyond hydrogen-evolution or oxygen-evolution potential, respectively, hydrogen or oxygen bubbles can be observed. However, in recent years, a quite different type of gas evolution has been found, i.e., under a magnetic field vertical to electrode surface, at an electrode potential far away from the gas evolution potentials, microbubble formation has been observed in a tornado-like rotation called vertical magnetohydrodynamic (MHD) flow shown in Fig. 1. On a micro-disk electrode placed sufficiently away from large counter electrode, current lines take a radial distribution, so that under a vertical magnetic field, a rotational Lorentz force is induced, depending on the directions of the current and magnetic field. As a result, the solution over the electrode circulates to form a tornado-like rotation (vertical MHD flow), which is, as shown in Fig. 1, characterized by inner and outer regions. In the outer region, centrifugal force by the fluid rotation is balanced with radial pressure gradient, whereas in the inner region, tangential velocity of the rotation suffers a deceleration from the friction of the fringe and disk at rest, which decreases the centrifugal force materially. The predominant radial pressure inevitably induces a secondary flow directed inwards, so that ionic vacancies produced at the electrode are carried toward the stagnation point at the center of the electrode, where they collide with each other to yield nanobubbles. Further collision of nanobubbles accelerates microbubble formation via. Ostwald ripening. Such bubbles are quickly conveyed upwards by the upward flow arising from the mass conservation of the secondary flow. As represented in Fig. 2, in ferrocyanide oxidation at a much more negative potential than oxygen-evolution potential, coalesced microbubbles have been first observed by Sugiyama *et al*.^3^. The same types of microbubbles independent of the gas evolution reactions have been continuously reported; in Figs 3 and 4, the images of microbubble coalescence in copper cathodic deposition and copper anodic dissolution are exhibited, respectively^4^,^5^. In all the cases, the gases inside the bubbles were determined as nitrogen gas experimentally dissolved in the solutions. As a result, next problem how such bubbles are formed was opened for us.

From about ten years ago, we have been studying ionic vacancies produced in electrode reactions. Though ionic vacancy in solid electrolyte is a popular point defect^6^,^7^,^8^,^9^, ionic vacancy in this case exists in a quite different environment, i.e., in liquid electrolyte solution, which is, as shown in Fig. 5, a negatively or positively polarized free vacuum space surrounded by oppositely charged ionic cloud, of which size in steady state is of the order of 0.1 nm^10^. By measuring the partial molar volume of ionic vacancy, the average radii of ionic vacancies having one and two unit charges were determined as 0.4 and 0.75 nm at 300 K^4^. The lifetime was measured as ca. 1 s at a room temperature by cyclotron MHD electrode^5^,^11^, which is composed of a pair of concentric circular cylinders partly exposed as electrodes, operating in a vertical magnetic field. Induced Lorentz force makes ionic vacancies together with electrolyte solution circulate along the cylindrical walls. Under low magnetic fields, due to low velocities, produced ionic vacancies become extinct, whereas under high magnetic fields, owing to high velocities, they can return to the same positions of the electrodes. As a result, the wall surfaces turn from rigid to free without friction. Using the difference of current response between the mass transfers on rigid and free electrode surfaces, the lifetime have been measured. However, the collision between ionic vacancies decreased the lifetime down to ca. 10 ms, which indicates that nanobubbles^12^,^13^,^14^,^15^,^16^,^17^,^18^,^19^ arise from the collision of ionic vacancies. Then, the formation process of nanobubbles from ionic vacancies was theoretically examined^20^, and it was found that dissolved gas goes into nanobubbles together with the transition from ionic vacancies to nanobubbles. Though a single nanobubble is too small to observe, their coalescence or conversion to microbubbles by Ostwald ripening can be optically detected. Actually, as have been shown in Figs 2, 3, 4, vertical MHD flows successfully provided the collisions between ionic vacancies. These results strongly suggest that ionic vacancies are stably created with extremely long lifetimes in any electrode reaction. However, the most essential problem is still unsolved, i.e., what kind of mechanism works for the production of ionic vacancy ? 

Therefore, the purpose of this paper is to theoretically clarify how ionic vacancy is produced in electrode reaction.

## Theory

According to Frank-Condon principle, electronic transition or transfer in step of electrode reaction is so fast that it can be regarded as taking in a stationary nuclear framework^21^, which therefore gives rise to the following problems of momentum and electric charge conservations.

### Momentum conservation in electrode reaction

As mentioned by Kittel^22^, the momentum of a free electron 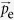 in common metal is related to the wavevector 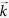 by 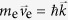, where *m*_e_ and 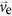 are the mass and velocity of electron, respectively, and *ħ* (=1.05457 × 10^−34^ Js) is the rationalized Planck constant. In an electric field 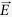 and magnetic field 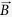, the external force 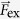 on an electron of charge −e (= −1.602177 × 10^−19^C) is 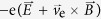, so that Newton’s second law of motion becomes


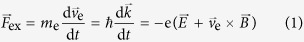


When the force is not applied, Fermi sphere encloses the occupied electron orbitals in 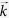 space in the ground state of electron gas. The net momentum is zero, because for every orbital 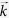, an occupied orbital at 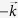 always exists. That is, in equilibrium, there is no net transfer of electron momentum. Under the influence of a constant force 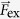 acting for a time interval *t*, every orbital has its 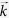 vector measured by 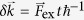. This is equivalent to a displacement of the whole Fermi sphere by 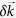. Because of the collision of electron with impurities, lattice imperfections and phonons, the displaced sphere may be maintained in a steady state in an electric field 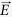. If the collision time is *τ*, the displacement of the Fermi sphere in the steady state is given by 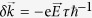. In view of Ohms law, using the electrical conductivity of copper at 295 K, *σ* = 5.88 × 10^7^ (Ωm)^−1^ and electron concentration^22^ *n*_e_ = 8.45 × 10^28^ m^−3 ^ as well as the stationary mass of electron *m*_e_ = 9.10956 × 10^−31^ kg, we can derive 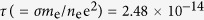 s. Therefore, the displacement 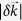 in this case is estimated as the order of 10^3^ m^−1^ for an electric field of the order of 10^2^ Vm^−1^. On the other hand, the Fermi wavevector of copper at room temperature^22^ is given by *k*_*F*_ = 1.36 × 10^10^ m^−1 ^. That is, *k*_F_ is much larger than 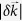, so that the electron momentum is regarded constant even when current flowing. Therefore, a free electron of copper at room temperature retains the momentum,


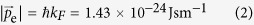


This means that the Fermi energy 
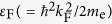
 also remains constant, i.e., for copper free electron, 7.00 eV at room temperature^22^.

In the same way, the electron velocity 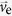 is interpreted as the incremental drift velocity, 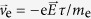, so that 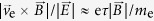 is derived. In the present case, 
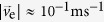
 and 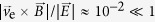 at a 10 T magnetic field are estimated. Namely, the contribution of magnetic field to the external force in Eq. 1 can be disregarded.

**Figure 6:**
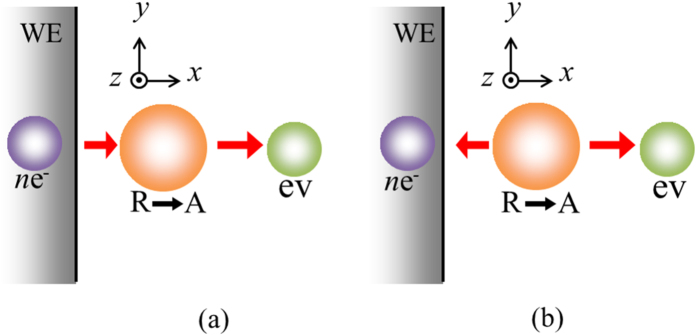
Emission of embryo vacancy in electrode reaction. (**a**) Cathodic reaction; (**b**) Anodic reaction. *n*e^−^, electrons transferring in the reaction; *R*, reactant; *A*, activated complex; ev, embryo vacancy; WE, woking electrode.

When the electrode potential is shifted to overwhelm the barriers such as work function and surface potential, electrode reaction occurs in keeping the same situation of free electron. In the reaction, Newton’s second law is again applied to the electrode system containing electrons, reactant, activated complex and original (embryo) vacancy shown in Fig. 6a,b, i.e.,


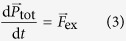


where 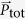 and 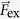 are the total momentum of the four species and the total external force imposing to them, respectively. Equation 3 is integrated from the initial state at a time *t*_ini_ to the activated state at a time *t*_act_ in the following,


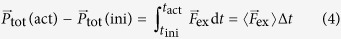


where 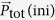 and 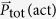 are the total momentums in the initial and activated states of the reaction, respectively, 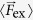 is the average external force for a time interval of Δ*t* defined by *t*_act_ − *t*_ini_. In an electric double layer during electrode reaction especially in case of diffusion control, the electric field 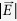
23 is estimated at most 10^7 ^Vm^−1 ^, so that in view of Eq. 1, 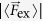 is estimated as 10^−12^ N. On the other hand, by using the electron velocity *v*_F_(=*ħk*_F_/*m*_e_) at the Fermi surface of copper at room temperature (=1.57 × 10^6^ ms^−1^)^22^, the time interval Δ*t* for an electron to transfer a distance of 1 nm is estimated of the order of 10^−15^ s.

Because Δ*t* is extremely short, the impulse 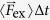 in Eq. 4 becomes the order of 10^−27^ Jsm^−1^, negligibly small in comparison with the momentum of the electrons, 10^−24^ Jsm^−1^. As a result, the total momentum involving electrons is conserved between the initial and activated states.


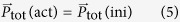


Then, in accordance with Frank-Condon principle, the reactant and activated complex are stationary during electric charge transfer, so that the momentums of the reactant and activated complex are equalized to zero. Namely, for cathodic and anodic reactions, the following momentum conservation between electrons and original embryo vacancy must be fulfilled.

For cathodic reaction, as shown in Fig. 6a, in the initial state, electrons transfer from the electrode to a stationary reactant in the *x*-direction, whereas in the activated state, from a stationary activated complex to the solution, an embryo vacancy is emitted in the same direction as the electrons. In view of one-dimensional process in *x*-direction, the initial and activated momentums are expressed by


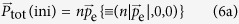



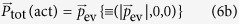


where 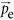 and 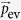 are the momentums of the electron and the embryo vacancy, and *n* is the electron number transferring in the reaction process. Using the momentum conservation equation in Eq. 5, we can calculate the momentum of the embryo vacancy equal to that of the electrons.


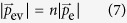


In anodic reaction, as shown in Fig. 6b, a stationary reactant in the initial state is decomposed into a stationary activated complex, electrons and an embryo vacancy in the activated state, where it should be noted that the electrons transfer in the opposite direction to that of the embryo vacancy, i.e.,


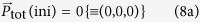



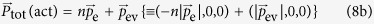


Substituting Eqs 8a and 8b into Eq. 5, we obtain the same equation as Eq. 7. Namely, whether the reaction is anodic or cathodic, an embryo vacancy with the same momentum as that of electrons transferring in electrode reaction is always created.

In the solution, the embryo vacancy behaves as a classical Boltzmann particle, which means that the average kinetic energy is equal to 3*k*_B_*T*/2, i.e.,


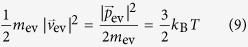


Substituting Eq. 7 into Eq. 9, we have the equation of the mass of the embryo vacancy,


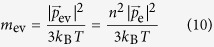


and the average velocity is given by


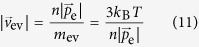


For simplicity, from now on, as for the numerical calculation concerning the momentum of ionic vacancy, we treat the case of a single electron transfer *n* = 1. Here, Boltzmann constant *k*_B_ = 1.38062 × 10^−23^ JK^−1^ and *T* = 295 K are used, so that inserting Eq. 2 into Eqs 10 and 11, we have *m*_ev_ = 1.69 × 10^−28^ kg and 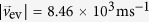. Comparing *m*_ev_ with the stationary masses of proton (1.67261 × 10^−27^ kg) and electron (9.10956 × 10^−31^ kg), we can see that *m*_ev_ takes a middle value between them, i.e., about 1/10 of that of proton and 100 times larger than that of electron. 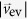 is about 1/100 of Fermi velocity of electron at room temperature (1.57 × 10^6^ ms^−1^)^22^ and about 100 times higher than that of solution particle such as water molecule. Since ionic vacancy is a classical Boltzmann particle, as will be discussed later, the small mass of the original embryo vacancy implies that the size is much smaller than those of other solution particles.

## Conservation of electric charge in electrode reaction

**Figure 7:**
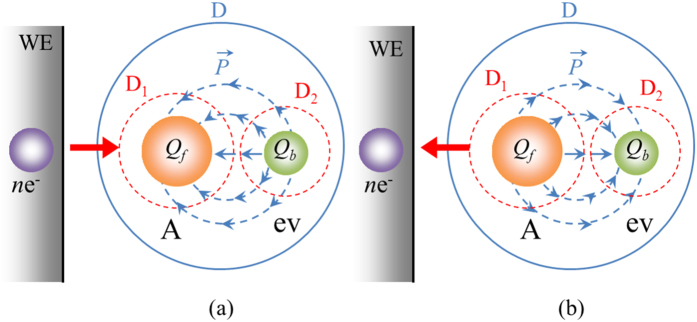
Charge transfer process by dielectric polarization (**a**) Cathodic reaction; (**b**) Anodic reaction. A, the activated complex; ev, embryo vacancy; *Q*_f_, the free electric charge by electron transfer; *Q*_b_, the bounded electric charge by dielectric polarization. The head and tail of an arrow implies plus and minus parts of the electric polarization vector 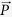, respectively.

As have been shown in the former section, for the momentum conservation in electronic transfer, Frank-Condon principle plays an important role in creating original embryo vacancy. Next problem to solve is therefore how about the conservation of electric charge. As have been initially mentioned, electronic transfers are so fast that they can be regarded as taking in a stationary nuclear framework. Based on the classical electromagnetic theory, this means that as shown in Fig. 7a,b, in a domain D enclosing a system of reactant, activated complex, electron and embryo vacancy, the electric field is invariable between the initial and activated states, i.e.,


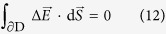


where Δ denotes the difference between the activated and initial states, i.e., 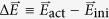, and 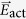 and 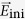 are the electric fields in the activated and initial states, respectively. ∂D is the closed surface of the domain D, and 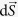 is the surface element vector. The electric change concerning electron transfer therefore must be completed within the D. Equation 12 is the Frank-Condon principle expressed by Maxwell equations.

The law of indestructibility of electricity must be fulfilled during the electric charge transfer between electrode and reactant in the following,


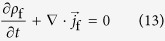


where 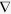 is nabla, *ρ*_f_ is the free electric charge density from transferring electrons, 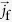 is the resulting free current density, and subscript f implies the free electric charge. In accordance with the discussion in the former section, Eq. 13 is integrated from the initial state at a time *t*_ini_ to the activated state at a time *t*_act_. Namely, at a time interval 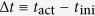, the free electric charge *Q*_f_ enters to or leaves from the domain D enclosing the reactant and activated complex, so that integrating 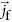 over the surface of D, we obtain *Q*_f_.


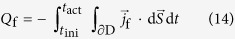


where the minus sign is added to the right hand side of Eq. 14 because the surface element vector 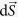 is defined positive when directed from the inside of the domain to the outside. Using Gauss’s theorem, we get


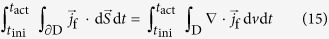


where 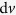 is the volume element of the domain 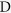. Then, substituting Eq. 13 into Eq. 15, and inserting the resulting equation to Eq. 14, we have


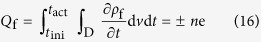


where the sign ± corresponds to oxidation and reduction, respectively. *n* and e are the charge number transferring in the reaction and the unit electric charge, respectively.

An activated complex is formed by the introduced charges, which are, based on Frank-Condon principle, regarded as free charges independent of the nuclear framework. In accordance with the activation, the electric flux density 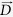 in the domain D is therefore changed, and the embryo vacancy created from the momentum conservation is in turn dielectrically polarized, which is expressed by the electric polarization 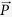, and the relationship between. 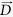, 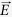 and 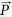 is given by


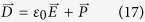


where *ε*_0_ is the absolute permittivity of free surface. Because the change in electric field concerning the electron transfer must be completed within the domain D (Eq. 12), as shown in Fig. 7a,b, the domain D can separately converge to the domains D_1_ of the activated complex and D_2_ of the embryo vacancy, respectively. By substituting Eq. 17 into Eq. 12, the differences between the initial and activated states of 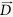 and 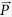 are equalized with each other, i.e.,


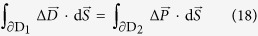


As mentioned above, the electric flux density 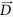 arises from the free electric charge introduced to the reactant, so that the following relationship with the free electric charge density *ρ*_f_ is fulfilled.


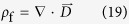


Therefore, by means of Gauss’s theorem, the free electric charge by the charge transfer is represented by


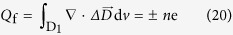


On the other hand, the electric polarization 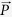 emerges from the induced electric charge bounded at the inner wall of the embryo vacancy, being expressed by


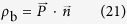


where *ρ*_b_ is the polarized surface charge density appearing on the inner wall, 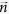 is the normal unit vector of the inner wall, and subscript b implies the bounded charge on the inner wall. In accordance with Fig. 7a,b, the polarization charge bounded in the embryo vacancy *Q*_b_ is obtained by the surface integration of the inner wall, which is equal to the surface integration of the domain D_2_.


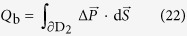


In view of Gauss’s theorem, substituting Eqs. 20 and 22 into Eq. 18, we get


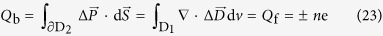


Namely, in an embryo vacancy, electric charge of the same value and sign as the transferring electric charge in the reaction is induced; in cathodic reduction, vacancies with negative charges are polarized, whereas vacancies with positive charges are induced in anodic oxidation. From momentum and electric charge conservations, redox electrode reactions are thus be described by


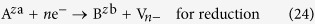



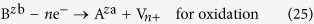


where A^*z*a^ and B^*z*b^ are ionic species, *n*e^−^ is the transferring electrons in the reactions. *V*_*n*−_ and *V*_*n*+_ are the ionic vacancies negatively and positively charged with *n* unit charges.

## Conservation of energy in equilibrium state

The equilibrium condition between ionic species A^*z*a^ and B^*z*b^ is without vacancy production written by


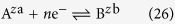


The corresponding potential relationship is expressed by


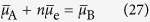


where 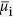 denotes the electrochemical potential of the species i.

In common metals, 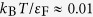 is fulfilled at room temperature^22^, so that electrochemical potential of electron 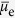 is closely equal to Fermi energy *ε*_F_, which is defined as the energy of the topmost filled level in the ground state of the N electron system. The Fermi sphere encloses the occupied electron orbitals in space in the ground state, where the net momentum is zero, because for every orbital 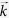, there is an occupied orbital at 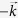. This indicates no electron momentum transfer, i.e., no ionic vacancy production in equilibrium state. If permitting the production, according to Eqs. 24 and 25, ionic vacancies would be created without limitation. In other word, in equilibrium state, there is no actual electron transfer between electrode and active species.

As discussed above, embryo vacancy is thought to be only a vacuum void with polarized electric charges, so that a stationary vacancy has no mass. However, when a vacancy moves in solution, we can easily imagine a solution particle of the same size as the vacancy oppositely moving, i.e., a moving vacancy obtains mass estimated by the average solution density. This means that a steady-state vacancy possesses the following average mass, which is given by


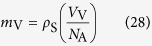


where *ρ*_S_ is the density of electrolyte solution, equalized to that of water, and *V*_V_ is the partial molar volume of steady-state ionic vacancy. For water at 295 K, *ρ*_S_ ≈ 10^3^ kgm^−3^ is obtained. If in the initial stage of the vacancy formation, the volume is also proportional to the mass, the volume of an embryo vacancy with a single unit charge is given by


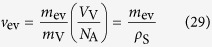


Then, the radius is written by


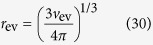


As have been mentioned in the section “Momentum conservation in electrode reaction”, in the simplest case of a single electron transfer, the volume is obtained as *v*_ev_ = 1.69 × 10^−31^ m^3^, so that the radius is *r*_ev_ = 7.36 × 10^−11^ m.

On the other hand, the formation period *τ*_ev_ of the embryo vacancy is estimated by


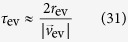


Equation 31 leads to *τ*_ev_ ≈ 1.76 × 10^−14^ s, which is 10 times longer than ordinal electron transfer time^24^,^25^,^26^, 10^−15^ s, but much shorter than the collision period of thermal particles^21^, 10^−10^ s. The period longer than that of electronic transfer ensures the formation of embryo vacancy with *n* unit charges. From these results, it can be concluded that immediately after electron transfer to or from a reactant in solution phase, with keeping the polarized electric charge, an embryo vacancy is created. Then, according to the thermodynamic process shown in ref. 10, it develops into a steady-state vacancy of ca. a 10^−10^ m radius by the coulombic attraction from oppositely charged outer ionic cloud. These results suggest that conventional theories of electrode reactions involving no ionic vacancies are insufficient with regard to conservations of momentum and electric charge. Detailed conclusions in summary are as follows: (1) In conventional electrode reaction theories, the exact examination of equation of motion describing momentum balance in electron transfer has not yet been done. The rigid application of Newton’s second law of motion indicates that the momentum difference of a reaction system between activated and initial states is equal to the impulse at electron transfer. The force imposed to the system is attributed to the electric field in electric double layer, which is sometimes large, however, owing to quite short period of electron transfer, the impulse is negligible comparing with the momentums themselves. This fact leads to an important conclusion that in electron transfer, the momentum of reaction system must be conserved. On the other hand, Frank-Condon principle requires that reactant and activated complex are stationary during the charge transfer, i.e., the momentums of reactant and activated complex are equal to zero. Therefore, in the case of single electron transfer, the momentum of an electron must be balanced by something else, i.e., that of ionic vacancy, which always gives rise to the emission of initial (embryo) vacancy. Its moving mass takes a middle value between the stationary masses of proton and electron, i. e., 1.69 × 10^−28^ kg, and the velocity is derived as 1.57 × 10^6^ ms^−1^, so that the average radius and the formation time are estimated as 7.36 × 10^−11^ m and 1.76 × 10^−14^ s, respectively. (2) Frank-Condon principle also demands the conservation of electric charge; since in electron transfer, the reaction system retains a stationary nuclear framework, the electric change during electron transfer must be completed within the system. Maxwell equations suggest that the electric charges introduced by electron transfer must be compensated by the dielectric polarization on the inner wall of ionic vacancy, i. e., ionic vacancy acquires the same charges as transferring in reaction. (3) At an equilibrium state, there is no vacancy production. If permitting such production, ionic vacancies would be created without limitation. This implies that in equilibrium, there is no actual electron transfer between electrode and active species. (4) The initial embryo vacancy emitted to solution side develops up to a steady-state vacancy with a 10^−10 ^m radius by the thermal motion of solution particles. Under a vertical magnetic field, vortex rotation called vertical MHD flow provides a collision field for steady-state vacancies. After collision, nanobubbles including dissolved gas are evolved, and furthermore, coalescence by Ostwald ripening converts the nanobubbles into microbubbles. (5) Since individual reaction processes are unrelated, the same discussion can be applied not only to electrode reaction, but also to all the chemical reactions transferring electrons and other particles such as photon. Namely, vacancy production in chemical reaction is a universal phenomenon, and must be always considered for the equation of motion in chemical reaction.

## Methods

**Figure 8:**
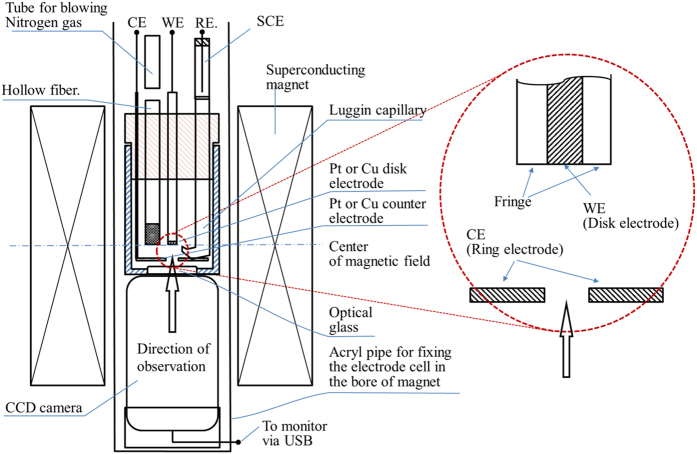
Experimental setup for obeserving electrode surface during eletrochemical reaction. WE, Working electrode; CE, Counter electrode.

The experimental apparatuses for observing microbubbles were consisted of home-made electrode cells and CCD cameras (Dino-Lite Premier2 S-DINOAD7013MT, AnMo Electronics Corp.) equipped with white LED lights which have the flame rate of 10 fps. The cells have flat bottoms covered with optical glasses, through which the surfaces of downward-oriented micro-disk (working) electrodes were optically observed. On the downsides of the electrodes, much larger circular (counter) electrodes were placed sufficiently away from the working electrodes. Through the holes of the counter electrodes, the surfaces of the working electrodes were observed by the CCD cameras. Saturated calomel electrodes (SCE) (International Chemistry Co., Ltd.) as reference electrodes were inserted in the neighborhood of the working electrodes. The CCD cameras were capable of a magnification power of ca. 200, connected with personal computers for *in situ* monitoring of the bubble evolution. The whole apparatuses were settled in the bore space (with an upward-oriented magnetic field) of the 40T hybrid magnet at the high magnetic field center, NIMS, Tsukuba Japan or the 15T cryocooled superconducting magnet at the High Field Laboratory for Superconducting Materials, IMR, Tohoku University. The apparatus including superconducting magnet is exhibited in Fig. 8. Experiments were performed in the following three cases; for ferricyanide/ferrocyanide redox reaction, platinum electrodes were used in a 100 mol m^−3^ K_3_ [Fe(CN)_6_] + 300 mol m^−3^ K_4_[Fe(CN)_6_] solution with a supporting electrolyte of 500 mol m^−3^ KCl. Under a magnetic flux density of 8 T, after 3 min. reduction at an overpotential of −200 mV (+230 mV vs. NHE), for ferrocyanide oxdation, the electrode potential was swept in a rate of 1 mV s^−1^ up to +400 mV (+830 mV vs. NHE)^3^. Then, for copper electrodeposition, copper electrodes were employed in a 30 mol m^−3^ CuSO_4_ solution with a supporting electrolyte of 100 mol m^−3^ H_2_SO_4_. Under a 8 T magnetic flux density, electrode potential was swept in a rate of 1 mV s^−1^ from the rest potential (+269 mV vs. NHE) to cathodic side down to −300 mV (−31 mV vs. NHE)^4^. Finally, anodic dissolution of copper electrode was carried out in 10 and 30 mol m^−3^ CuSO_4_ solutions containing a 100 mol m^−3^ H_2_SO_4_ supporting electrolyte. At 8 T, copper electrode was anodically dissolved at a constant overpotential of 150 mV (400 mV vs. NHE)^5^. Prior to experiment, for the evacuation of dissolved oxygen, nitrogen gas bubbling was performed. Then during the experiment, the gas was continuously supplied via a hollow fiber filter (M40-200, Nagayanagi Co., Ltd.) at atmospheric pressure.

## Additional Information

**How to cite this article**: Aogaki, R. *et al*. Origin of Nanobubbles Electrochemically Formed in a Magnetic Field: Ionic Vacancy Production in Electrode Reaction. *Sci. Rep.* **6**, 28927; doi: 10.1038/srep28927 (2016).

## Figures and Tables

**Figure 1:**
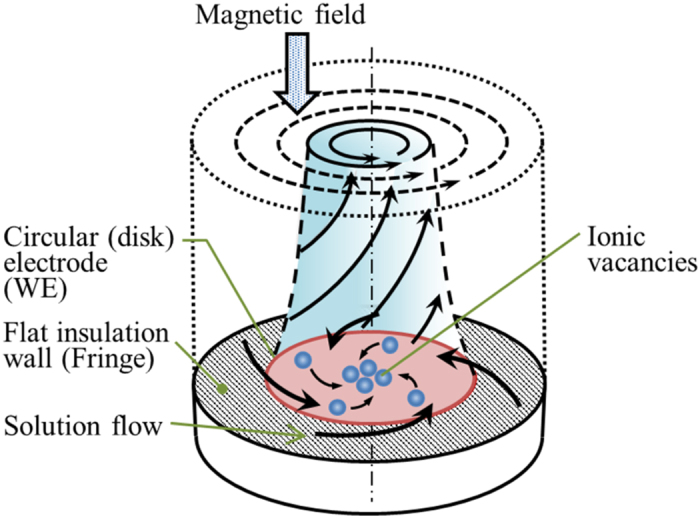
Vertical MHD flow for anodic reaction (Modified^3^). Small blue circles indicate ionic vacancies.

**Figure 2:**
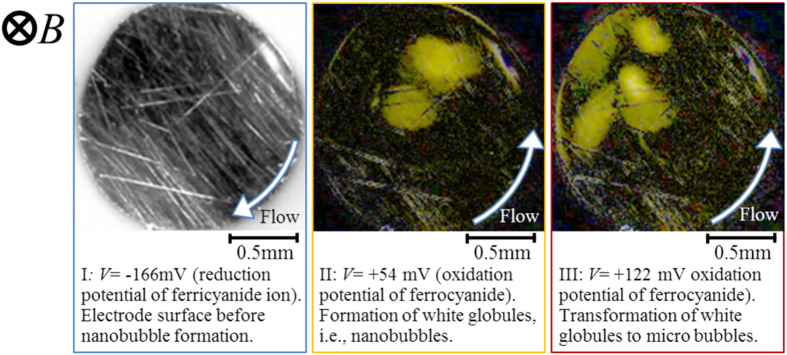
Microbubble evolution on the electrode in ferrocyanide oxidation under a 8 T magnetic field (Modified^3^). *V*, overpotential from the rest potential (*V* = −166 mV, +54 mV, +122 mV correspond to the electrode potentials, *E* = +264 mV, +484 mV, +552 mV vs. NHE, respectively); 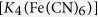, 300 molm^−3^; 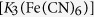, 100 molm^−3^; [KCl], 500 molm^−3^.

**Figure 3:**
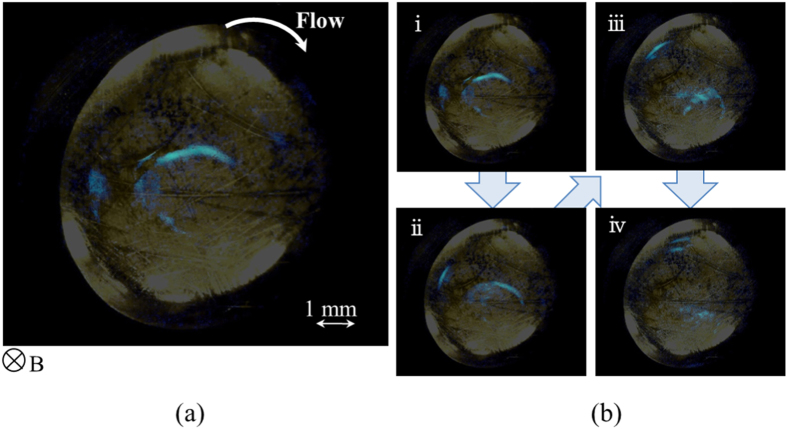
Microbubble evolution in copper cathodic deposition (Modified^4^). *B*, magnetic flux density. [CuSO_4_], 30 molm^−3^; [H_2_SO_4_], 100 molm^−3^. (**a**) Electrode surface at V = −144 mV (+125 mV vs. NHE); (**b**) Continuous images of the globule motion at a 70 ms interval. The observed angular velocity was 6.67 s^−1^, which is shown in the form of arrow in (**a**). For clear visualization, the images of the globules are painted in blue.

**Figure 4:**
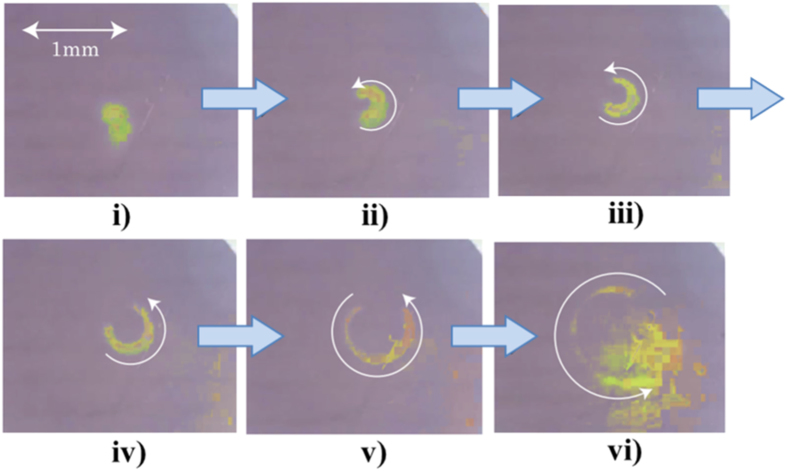
Continuous images of microbubble evolution in copper anodic dissolution under a 8T magnetic field taken at a 0.33 s interval (Modified^5^). *V* = +150 mV (+400 mV vs. NHE); [CuSO_4_], 30 mol m^−3^; [H_2_SO_4_], 100 mol m^−3^.

**Figure 5:**
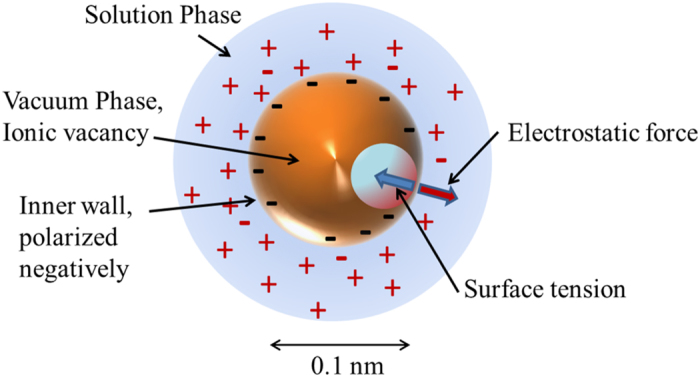
Ionic vacancy negatively polarized in a steady state.
